# Retinal Damage in Multiple Sclerosis Disease Subtypes Measured by High-Resolution Optical Coherence Tomography

**DOI:** 10.1155/2012/530305

**Published:** 2012-07-25

**Authors:** Timm Oberwahrenbrock, Sven Schippling, Marius Ringelstein, Falko Kaufhold, Hanna Zimmermann, Nazmiye Keser, Kim Lea Young, Jens Harmel, Hans-Peter Hartung, Roland Martin, Friedemann Paul, Orhan Aktas, Alexander U. Brandt

**Affiliations:** ^1^NeuroCure Clinical Research Center and Experimental and Clinical Research Center, Charité University Medicine Berlin/Max Delbrück Center for Molecular Medicine, 10117 Berlin, Germany; ^2^Institute for Neuroimmunology and Clinical Multiple Sclerosis Research (inims), University Medical Center Hamburg-Eppendorf, 20251 Hamburg, Germany; ^3^Department of Neuroimmunology and Clinical Multiple Sclerosis Research, Neurology Clinic, University Hospital Zurich, 8091 Zurich, Switzerland; ^4^Department of Neurology, Medical Faculty, Heinrich-Heine-University Düsseldorf, 40225 Düsseldorf, Germany; ^5^Clinical and Experimental Multiple Sclerosis Research Center, Charité University Medicine Berlin, 10117 Berlin, Germany

## Abstract

*Background.* Optical coherence tomography (OCT) has facilitated characterisation of retinal alterations in MS patients. Only scarce and in part conflicting data exists on different MS subtypes. *Objective.* To analyse patterns of retinal changes in different subtypes of MS with latest spectral-domain technology. *Methods.* In a three-centre cross-sectional study 414 MS patients and 94 healthy controls underwent spectral-domain OCT examination. *Results.* Eyes of MS patients without a previous optic neuritis showed a significant reduction of both retinal nerve fibre layer (RNFL) thickness and total macular volume (TMV) compared to healthy controls independent of the MS subtype (*P* < 0.001 for all subtypes). RNFL thickness was lower in secondary progressive MS (SPMS) eyes compared to relapsing-remitting MS (RRMS) eyes (*P* = 0.007), and TMV was reduced in SPMS and primary progressive MS (PPMS) eyes compared to RRMS eyes (SPMS: *P* = 0.039, PPMS: *P* = 0.005). Independent of the subtype a more pronounced RNFL thinning and TMV reduction were found in eyes with a previous optic neuritis compared to unaffected eyes. *Conclusion.* Analysis of this large-scale cross-sectional dataset of MS patients studied with spectral-domain OCT confirmed and allows to generalize previous findings. Furthermore it carves out distinct patterns in different MS subtypes.

## 1. Introduction

Multiple sclerosis is an inflammatory and neurodegenerative disorder of the central nervous system that leads to a progressive axonal loss and degeneration of neurons. Whereas a vast majority of MS patients present with a relapsing-remitting disease course (RRMS) that may subsequently transforms into secondary progressive MS (SPMS), a smaller portion of patients shows a progressive course (PPMS) from the very beginning of the disease [1]. 

Optical coherence tomography (OCT) is an increasingly recognized, noninvasive tool in MS imaging that allows cost-effective investigation of the retina [2] in a disease in which pathology of the anterior visual system is common. Over the recent past, OCT has emerged as a potential marker of axonal retinal degeneration in MS patients [3]. Of note, an increasing number of studies have consistently shown an association between OCT measures of retinal atrophy and markers of degeneration derived from either structural magnetic resonance imaging or MR spectroscopy (MRS) studies [4–11] and between OCT measures and functional visual parameters as well as physical disability and cognitive performance [12–17]. Retinal nerve fibre layer (RNFL) thickness and total macular volume (TMV) are the most frequently investigated OCT parameters. They provide a unique opportunity to quantify the integrity of nonmyelinated axonal tissue (RNFL) as well as all retinal layers (TMV), including cellular segments, by a noninvasive imaging technique. RNFL and TMV reduction can be detected in eyes with (MS-ON) or without a previous history of optic neuritis (MS-NON) applying different OCT devices [15, 18–29]. Data on distinct differences in OCT findings between MS subtypes are scarce, and the results are at least in part conflicting. In general, cross-sectional studies show a more profound thinning of RNFL in progressive forms of MS compared to RRMS patients. However, it remains unresolved to date whether these differences are a genuine effect of the disease subtype *per se* or rather a function of disease duration, the number of optic neuritis (ON) episodes in the patients' history, or disease severity [30–34]. 

Most OCT studies previously performed in MS applied time-domain technology. Only very recently spectral-domain technology became available which enables imaging at substantially higher resolution without an increase in scanning time [16, 22, 34–38]. Here we report results from the largest multicentre cohort of MS patients and healthy controls (HC) thus far, studied in three dedicated MS centres in Germany, applying latest high-resolution spectral-domain OCT (SD-OCT) technology. In this large cohort, we reliably identified patterns of RNFL thinning and TMV reduction among different MS subtypes both with and without a history of ON when controlling for disease duration and severity, age, and gender.

## 2. Materials and Methods

### 2.1. Patients and Controls

414 patients and 94 healthy controls were recruited in three dedicated MS units in the respective outpatient clinics at the Charité University Medicine Berlin (NeuroCure Clinical Research Center (NCRC)), at the Department of Neurology of the Heinrich-Heine University Düsseldorf (UKD) and in Hamburg (Institute for Neuroimmunology and Clinical MS Research (inims)). Data from a subgroup has previously been reported in Brandt et al. [23]. Inclusion criteria were age between 18 and 60 years and a definite diagnosis of MS according to the revised 2005 McDonald criteria [39]. MS subtype classification in RRMS, SPMS, or PPMS was based on the clinical course as assessed by the treating physician using Lublin criteria [40]. A history of ON had to be clearly determinable either by existing medical records, a VEP suggestive of optic neuritis, or by patient self-reports. Only eyes in which a history of ON could either be confirmed by clinical records or ruled out were subsequently included in the analysis. Patients with a refractive error of ≥5.0 diopters or with a history of eye disease that may impact significantly on OCT measures (e.g., glaucoma, retinal disease, retinal surgery, and diabetes) were excluded. Other exclusion criteria were acute optic neuritis or any other acute relapse or steroid treatment less than six months prior to OCT assessment as well as any other neurological disease with possible ocular manifestations. Disease duration was calculated as time since diagnosis in months.

Participants were assessed in a clinical examination under supervision of board certified neurologists within 6 months of OCT. Extended disability status scale (EDSS) was calculated according to the current guidelines [41]. Time since diagnosis was determined by reviewing patients' medical records. Healthy control participants were recruited from the medical staff, patients' relatives, and other volunteers.

A total of 937 eyes (754 MS eyes and 183 HC eyes) were finally included, 79 eyes were excluded from the analysis either due to poor scan quality or incomplete clinical data, in detail missing data on history of ON.

### 2.2. Ethics

The study protocol was approved by the local ethics committee and was conducted in accordance with the Declaration of Helsinki (1964) in its currently applicable version, the Guidelines of the International Conference on Harmonization of Good Clinical Practice (ICH-GCP) and applicable German laws. All participants gave written informed consent.

### 2.3. Optical Coherence Tomography

Participants underwent SD-OCT examination using the Heidelberg Engineering Spectralis SD-OCT (Heidelberg Engineering, Heidelberg, Germany). For both eyes of each participant, RNFL thickness around the optic disc was acquired using the 3.4 mm circle scan with the eye tracker system (TrueTrack) activated and the maximum number of averaging frames in ART-MEAN mode were tried to achieve. For assessing the macular volume two different scan protocols were used: the system built-in macula scan (25 B-scans, scanning angle = 15° × 15°, ART = 9) was performed at the Hamburg and Düsseldorf sites, while for the macular volume determination at the NCRC a custom protocol (61 B-scans, scanning angle = 30° × 25°, ART = 13) was used. Irrespective of the macular scan type, the TMV was calculated using the device's software. All scans were performed by trained operators and were reviewed for sufficient signal strength, correct centring, and beam placement as well as segmentation. Only eyes that passed the quality review were included in the subsequent analysis.

### 2.4. Statistical Analysis

Group comparisons of demographic factors were analysed using Mann-Whitney *U* test (for age and EDSS) and Pearson's Chi-square test (for gender, alpha = 0.05). Within the MS cohort Spearman's Rho analysis was used for correlation of EDSS and disease duration. 

Generalized estimation equation (GEE) models accounting for within-subject intereye correlations were applied to test for differences of RNFL thickness and TMV between the study cohorts. GEE models were corrected for age and gender for the comparison of MS patients with HC and additionally for disease duration for MS subtype analysis. Associations of OCT with EDSS and regression analysis of disease duration with RNFL thickness and TMV were investigated with GEE models in a similar fashion. In all GEE models OCT measurements were included as the dependent variable. All statistical analyses were performed with R (R Version 2.12.2) including the *geepack* package for GEE models. Statistical significance was established at *P* < 0.05.

## 3. Results

### 3.1. Cohort Description

An overview of the demographic and basic clinical data including MS subtypes is given in Table 1. Healthy controls showed no significant gender differences to RRMS (Chi-square: *P* = 0.452) and SPMS (Chi-square: *P* = 0.186) patients, while gender composition of PPMS patients differed compared to HC (Chi-square: *P* = 0.010). Mean age of HC was significantly lower compared to all MS patients and all subtypes (Mann-Whitney *U* tests, *P* < 0.001 for all subtypes). RRMS patients were significantly younger than the progressive subtypes (Mann-Whitney *U* tests, *P* < 0.001 for SPMS and PPMS). As a consequence, age and gender were included as covariates in all GEE models. Time since diagnosis of RRMS and PPMS was significantly shorter compared to SPMS (Mann-Whitney *U* tests, *P* < 0.001 for both). Therefore, GEE models for MS subtype comparison were additionally adjusted for disease duration. Disease severity estimated by the EDSS was heterogeneous between MS subtypes as evaluated by Mann-Whitney *U* tests (RRMS versus SPMS: *P* < 0.001; RRMS versus PPMS: *P* < 0.001; SPMS versus PPMS: *P* = 0.03).

### 3.2. RNFL and TMV in MS-NON Eyes of Different MS Subtypes Compared to HC

**Figure 1:**
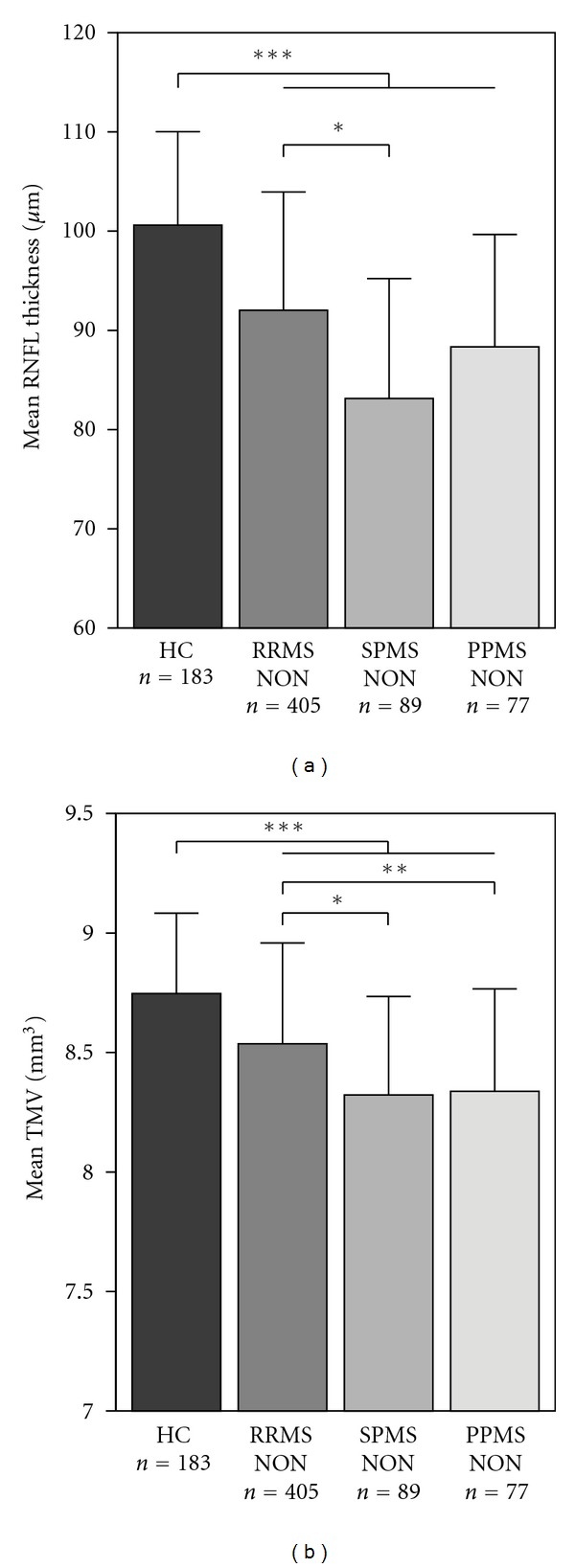
Mean retinal nerve fibre layer (RNFL) thickness (a) and mean total macular volume (TMV) (b) for the healthy controls (HC) and MS subtypes (RRMS, SPMS, and PPMS) without a history of optic neuritis (NON). Significant differences between the groups are indicated with *(*P* < 0.05), **(*P* < 0.01), and ***(*P* < 0.001), respectively.

For MS-NON eyes, differences in RNFL thickness and TMV compared to HC are given in Table 2. In summary, average peripapillary RNFL thickness was reduced in the pooled cohort of all MS patients' eyes and in all MS subtypes compared to HC (Figure 1(a)). Likewise, TMV was reduced for the pooled cohort of all MS patients' eyes and in all MS subtypes when compared to HC (Figure 1(b)). All alterations of RNFL and TMV in MS-NON eyes compared to control eyes showed strong statistical significance based on GEE models (Table 2). EDSS was inversely correlated with RNFL in case of all MS subtypes without a history of prior ON (RRMS-NON: *P* = 0.007; SPMS-NON: *P* = 0.034; PPMS-NON: *P* = 0.006). In contrast, the TMV was only significantly correlated with the EDSS in RRMS-NON eyes (*P* = 0.003), but not in SPMS-NON (*P* = 0.321) or PPMS-NON (*P* = 0.085).

### 3.3. Comparison of MS-ON Eyes with MS-NON Eyes and HC

**Figure 2:**
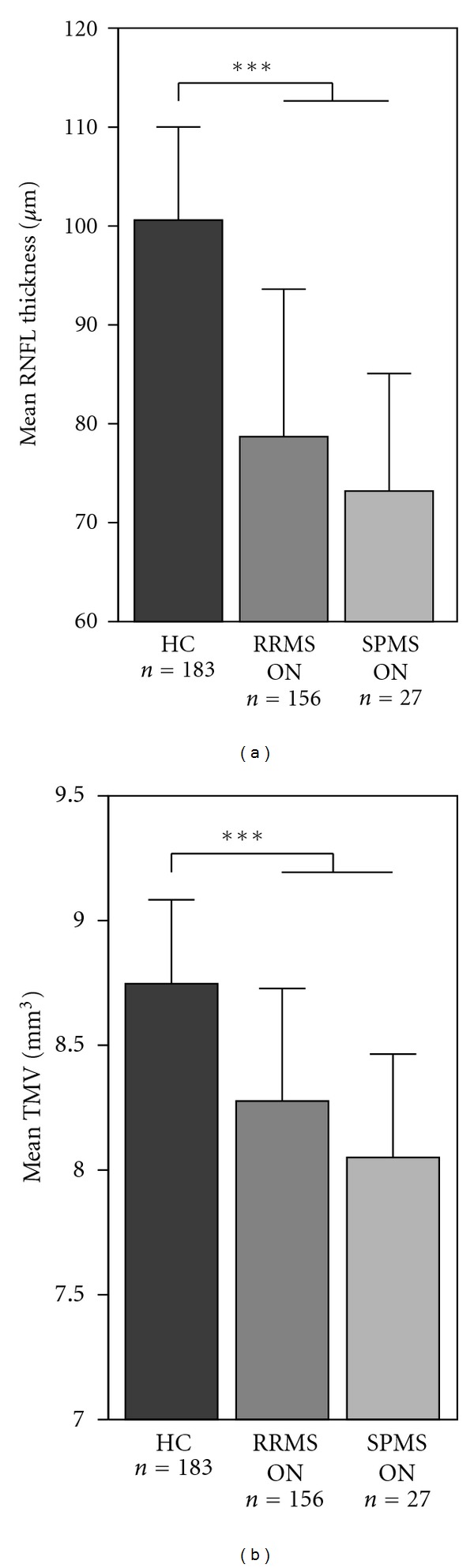
Mean retinal nerve fibre layer (RNFL) thickness (a) and mean total macular volume (TMV) (b) for the healthy controls (HC) and MS subtypes (RRMS, SPMS) with a history of optic neuritis (ON). Significant differences between the groups are indicated with *(*P* < 0.05), **(*P* < 0.01), and ***(*P* < 0.001), respectively.

Irrespective of the MS subtype, MS-ON eyes were significantly different from HC eyes for RNFL thickness and TMV (Figure 2) and showed a more pronounced RNFL thinning and TMV reduction when compared to MS-NON eyes (Table 3). RNFL thickness of RRMS-ON eyes and SPMS-ON eyes was significantly thinner compared to RRMS-NON or SPMS-NON eyes, respectively. The same was true for the TMV of ON-affected MS eyes, which was significantly reduced when compared to HC and to unaffected eyes of the same MS subtype (Table 3, Figure 2). The extent of RNFL thinning as well as TMV reduction was comparable between RRMS-ON and SPMS-ON eyes (RNFL GEE: *P* = 0.369; TMV GEE: *P* = 0.124). RRMS-ON eyes showed a significant inverse correlation between EDSS and RNFL (*P* = 0.012) while TMV did not reach significance (*P* = 0.085). For SPMS-ON eyes no significant correlation of EDSS with OCT parameters was found (RNFL: *P* = 0.169; TMV: *P* = 0.573).

### 3.4. Differences between Subtypes in MS-NON Eyes

Among MS subtypes, RRMS patients showed less RNFL thinning when compared to progressive subtypes (Table 2). When adjusting GEE models for age, gender, and disease duration, the only significant difference based on the mean total RNFL thickness was found between RRMS and SPMS patients, while patients with PPMS did not differ from either RRMS or SPMS (Table 4, Figure 1). As opposed to RNFL thickness a different pattern was obtained for TMV measures, in which a significant reduction was found for SPMS and PPMS when compared to RRMS patients. GEE models in which we additionally corrected for EDSS to account for different stages of disease severity did not show differences in RNFL thickness or TMV between MS subtypes (data not shown).

### 3.5. Association with Disease Duration and Yearly Atrophy Estimate

**Figure 3:**
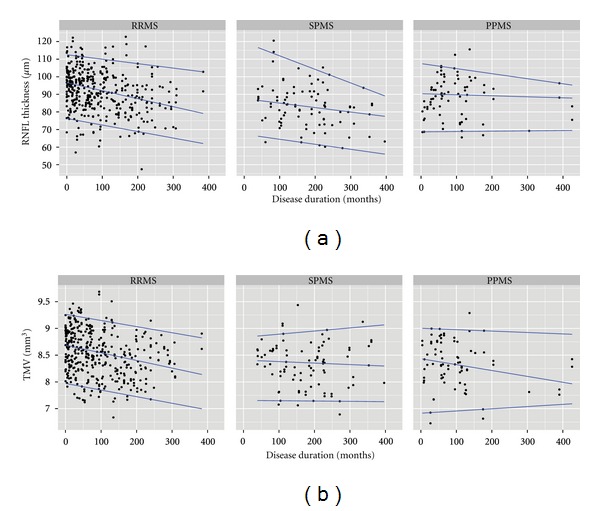
Association of RNFL thickness (a) and TMV (b) with disease duration for RRMS, SPMS and PPMS subtypes in eyes without previous optic neuritis. The blue lines indicate the 95%-, 50%- and 5%-quantiles.

GEE models were used to test for an association of disease duration and RNFL thickness or TMV in MS eyes. MS-NON eyes showed an association of RNFL thickness and TMV with disease duration in the pooled cohort of all MS subtypes (Table 5, Figure 3). This association was retained in RRMS and SPMS eyes only for RNFL thickness and only in RRMS eyes for TMV. In all MS subtypes the correlation between RNFL thickness and TMV and disease duration was lost in ON eyes (data not shown).

Based on the effect size of the association of disease duration and RNFL thickness or TMV we estimated RNFL thinning and TMV reduction per year of ongoing disease in MS-NON eyes only (Table 5). RRMS-NON eyes showed the strongest and highly significant yearly changes for RNFL thickness (−0.495 *μ*m/year) and TMV (−0.0155 mm^3^/year). Interestingly, the significant yearly RNFL thinning in SPMS-NON eyes (−0.464 *μ*m/year) was not concomitantly associated with a significant correlation in TMV change (−0.0016 mm^3^/year, *P* = 0.838). In contrast, PPMS-NON eyes showed a less pronounced yearly RNFL thinning (−0.105 *μ*m/year) but in relation a distinct reduction of TMV (−0.0111 mm^3^/year). However, correlations of RNFL and TMV with disease duration were not significant for PPMS-NON eyes.

## 4. Discussion

Here we present the largest ever performed cross-sectional study on retinal atrophy measures in MS subtypes applying latest SD-OCT technology. Groups of disease subtypes in our study were sufficiently large to contrast findings in ON versus ON-free eyes within subgroups. Hereby we show that both RNFL and TMV are reduced in MS-NON eyes versus HC when pooling all disease subtypes, but also when separately comparing disease subtypes (RRMS, SPMS, and PPMS) to HC. Not surprisingly and in accordance with previous studies, MS-ON eyes exhibited more severe RNFL and TMV damage than MS-NON eyes. Both findings have been previously described in a similar way by various groups so that the nature of our study appears to be largely confirmatory at first glance. However, our study has some methodological advances compared to previous works that have important implications for the interpretation of our and previous OCT findings. 

The large sample size of our study enabled a statistically robust comparison of various disease subgroups with the inclusion of possible confounding factors such as age, disease duration, and gender in the statistical models. In particular, we had larger numbers of patients in the progressive subgroups (65 SPMS, 41 PPMS) than any other previous study which allowed us to compare not only disease subtypes with HC but also with each other. This is of special interest against the background of the ongoing scientific debate on distinct pathogenetic mechanisms in, for example, PPMS versus RRMS. The subgroup comparisons revealed a significant reduction of RNFL thickness in SPMS patients versus RRMS after correction for age, gender, and disease duration and a significant reduction of TMV in both SPMS and PPMS patients versus RRMS. 

In contrast, most previous works had only small sample sizes, especially for progressive subtypes which may—besides considerable differences in the statistical models—at least partly explain the inconsistent findings. Pulicken et al. (number of subjects: 135 RRMS, 16 SPMS, 12  PPMS, and 47 HC) found only trends towards lower RNFL thickness values in progressive disease versus RRMS and no difference in TMV in progressive MS versus RRMS [30]. Henderson et al. (number of subjects: 27 SPMS, 23 PPMS, and 20 HC) reported a significant reduction of RNFL and TMV versus HC only in NON eyes from SPMS patients but not PPMS patients and no difference between PPMS and SPMS [31]. Siepman et al. (number of subjects: 26 RRMS, 10 SPMS, and 29 PPMS) could not detect differences between PPMS-NON eyes and the pooled RRMS/SPMS-NON eyes [33]. Serbecic et al. (number of subjects: 42 RRMS, 17 SPMS, and 59 HC) did not specifically address differences in OCT measures between disease subtypes [34] as did numerous other studies with highly heterogeneous patient populations (Gordon-Lipkin et al. [6], number of subjects: 20 RRMS, 15 SPMS, 5 PPMS, and 15 HC; Toledo et al. [12], number of subjects: 7 CIS, 36 RRMS, 3 SPMS, 3 PPMS, 4 progressive-relapsing, and 18 HC; Fisher et al. [15], number of subjects: 90 MS, 76 of whom RRMS, and 36 HC; Sepulcre et al. [7], number of subjects: 22 CIS, 28 RRMS, 5 SPMS, 6 PPMS, and 29 HC), either because of insufficient subgroup sample sizes or owing to the fact that the study had goals other than comparing disease subtypes. 

In line with several previous studies [16, 30, 31, 42, 43] we found higher RNFL measures in PPMS as compared to SPMS (88.4 *μ*m versus 83.1 *μ*m), which is in striking accordance with another study that also reported a difference of approximately 5 *μ*m between PPMS and SPMS-NON eyes (93.9 *μ*m versus 88.4 *μ*m) [31]. Although these differences were not significant in both studies, these findings may point to a more severe RNFL damage in SPMS as compared to PPMS, which is in line with the clinical features of PPMS with a lower proportion of visual loss, less frequent ON attacks, a predominant clinical involvement of the spinal cord, and smaller brain lesions as compared to SPMS [31, 44, 45]. However, in contrast to Henderson et al. we found like in SPMS a significant reduction of TMV in NON eyes of PPMS patients versus RRMS and HC, which may display the neurodegenerative component of PPMS concomitantly reflected through brain atrophy measures [46].

Regarding the comparison of RNFL measures in RRMS and SPMS patients, we made another interesting observation: as described previously by Costello et al. [32], differences between SPMS-NON and RRMS-NON eyes were about twice that of differences between SPMS-ON and RRMS-ON eyes (~20 *μ*m versus ~10 *μ*m in Costello's study, ~10 *μ*m versus ~5 *μ*m in our study). Costello et al. suggested that the impact of prior ON may outweigh the effects of disease subtype. 

Further limitation of most of the previous studies is the utilization of time-domain OCT devices (TD-OCT) that only allow for 2-dimensional retinal imaging, limiting its use especially in the demanding macular investigations. The newer high-resolution spectral-domain OCT allows spatial imaging of the retina thus potentially greatly increasing the accuracy and value of OCT in MS [35, 36, 47]. First studies have already applied SD-OCT with intraretinal segmentation [16, 22, 26, 29]. Interestingly, the recent work by Saidha et al. supports the finding of a more severe neuroaxonal retinal damage in SPMS as compared to PPMS; a separate analysis of the combined ganglion cell layer and inner plexiform layer measured by Cirrus SD-OCT in different MS subtypes showed lowest values in SPMS [16]. In contrast, another study by Albrecht et al. [29] applying manual segmentation on Spectralis SD-OCT showed reduced measures in the deeper inner nuclear layer of PPMS but not SPMS patients versus HC. We presume that the ability of SD-OCT to measure spatial scans (earlier TD-OCT had to interpolate macular volume by using 6 radial linear scans) will in future greatly increase the value of macular scans over the currently preferred peripapillary ring scans. In addition, spatial scans allow for correction of positioning errors after scan acquisition by limiting the analysed area to a subset of the actual scan. For example, the Cirrus SD-OCT uses a spatial scan for the peripapillary ring scan, allowing for subsequent correction of alignment errors, whereas the Heidelberg Engineering Spectralis facilitates an eye tracker function to correct for eye movements. In TD-OCT, incorrect placement of peripapillary ring scans accounts to a significant extent for a weaker inter-measurement reliability and cannot be corrected after the scan has been acquired [48]. Next to the ability to analyse all intraretinal layers, improved test/retest-reliability distinguishes SD-OCT from TD-OCT and makes it an ideal instrument for the use in a longitudinal setting where inter-measurement reliability is crucial [49].

The time course of RNFL thinning and TMV reduction by atrophy of different retinal layers—be it in the context of ON or independent thereof—is an essential characteristic in rating the usefulness of OCT as a potential marker of axonal loss in longitudinal clinical trials. For MS-ON eyes it has previously been shown that RNFL thinning occurs within the first 6 months after the ON attack [21, 50]. Overall little is known about temporal dynamics of retinal thinning in MS-NON eyes. Based on published data from cross-sectional studies in MS patients with different disease duration a rough estimate of the yearly atrophy rate appears to be around 1 *μ*m/year, which is ten times as much as what can be expected from normal ageing [3]. In previous cross-sectional studies significant inverse correlations of RNFL thickness and disease duration could be established by some authors [11, 15, 24], while others did not find a significant correlation [20, 31]. In PPMS, an MS subtype in which frequency of clinical attacks of ON is probably lowest, Henderson et al. [31] estimated an RNFL thinning of approximately 0.12 *μ*m (TMV reduction: 0.01 mm^3^) per year of disease, which is in good agreement with our results in PPMS eyes (RNFL thinning −0.105 *μ*m/year; TMV reduction: −0.011 mm^3^/year). Correlations of OCT measures of retinal atrophy and disease duration were not significant for PPMS patients in both studies. In case of RRMS and SPMS patients without ON we estimated higher yearly RNFL changes than for PPMS (nearly 0.5 *μ*m/year). It is, however, important to note that yearly atrophy rates are considerably lower than the optimized axial resolution of SD-OCT devices, which is approximately 4–6 *μ*m [51, 52]. This is of relevance in case OCT endpoints are taken into consideration for future clinical trials, for example, in proof-of-concept trials for neuroprotective agents. Depending on the disease subtypes, model timelines and sample sizes have to be planned accordingly.

In a first longitudinal OCT study by Talman et al. [53] a thorough examination of the time course of RNFL thinning in a mixed cohort of different MS subtypes was performed with TD-OCT (Stratus) revealing a yearly loss of approximately 2 *μ*m in MS-NON eyes (GEE: *P* < 0.001). The study included a preliminary sample size calculation (supplementary data of [53]) for future clinical trials that aimed to detect a 30% reduction in the proportion of eyes with an RNFL thinning greater than the test-retest variability of the Stratus OCT (6.6 *μ*m) over a follow-up period of 2-3 years. With a power of 80–90% and a type 1 error of 0.05, the authors' sample size calculation estimated roughly a number of 400–600 patients per group. The yearly loss of 2 *μ*m reported by Talman et al. from Stratus OCT is considerably higher than the yearly reduction of approximately 0.5 *μ*m calculated from our dataset. Discrepancies may derive from the differences in the devices applied (TD-OCT versus SD-OCT) and the fact that our calculation is based on cross-sectional data only.

 In sum, this study, based on a large SD-OCT data set, confirms previous data on neuroaxonal retinal damage in MS subtypes. At the same time, it extends previous findings by providing new insights into differences between MS-ON and MS-NON eyes in the various subgroups and—in addition—allowing for reliable correction for non-disease-related factors such as age, gender disease duration, and severity.

## Figures and Tables

**Table 1:** Demographic and clinical data of multiple sclerosis patients (MS) and healthy controls (HC). MS patients are subdivided in the subtypes relapsing-remitting MS (RRMS), secondary-progressive MS (SPMS), and primary-progressive MS (PPMS).

		HC	MS			
			RRMS	SPMS	PPMS	All MS
No. of Subjects	Total	94	308	65	41	414
	Berlin	63	95	22	8	125
	Hamburg	0	158	23	22	203
	Düsseldorf	31	55	20	11	86

No. of Eyes (eyes with ON)	Total	183	561 (156)	116 (27)	77 (0)	754 (183)
	Berlin	122	187 (77)	44 (15)	16 (0)	247 (92)
	Hamburg	0	270 (54)	35 (2)	41 (0)	346 (56)
	Düsseldorf	61	104 (25)	37 (10)	20 (0)	161 (35)

Gender	Male (%)	31 (33)	87 (28)	29 (45)	24 (59)	140 (34)
	Female (%)	63 (67)	221 (72)	36 (55)	24 (41)	274 (66)

Age (in years)	Mean (SD)	34.47 (10.25)	39.10 (9.50)	48.23 (6.11)	46.90 (7.10)	41.31 (9.59)
	Range	19–56	19–58	33–59	32–59	19–59

Disease duration (in months)	Mean (SD)	NA	91.05 (80.26)	186.15 (87.94)	100.02 (93.33)	106.87 (89.51)
	Range	NA	0–384	39–403	4–426	0–426

EDSS	Median	NA	2.0	5.5	4.0	2.5
	Range	NA	0-7	3-8	2-8	0-8

**Table 2:** OCT results for the subtypes of MS patients without a history of optic neuritis (NON). Total retinal nerve fiber layer (RNFL) thickness (in *μ*m) and total macular volume (TMV in mm^3^) are given as mean values with standard deviation (SD). Generalized estimation equation (GEE) models were used to compare the MS cohorts to healthy controls. GEE models estimate the effect size with standard error (SE) and the respective *P* value.

			GEE models comparing MS OCT parameters to the healthy control cohort						
				RNFL thickness			TMV		
	Total RNFL thickness mean (SD) [*μ*m]	TMV mean (SD) [mm^3^]	Effect	Effect size	SE	*P* value	Effect size	SE	*P* value
			Group	−9.571	1.177	<0.001	−0.235	0.043	<0.001
MS-NON (*n* = 571)	90.15 (12.27)	8.48 (0.43)	Age	−0.132	0.053	0.013	−0.006	0.002	0.002
			Gender	2.272	1.116	0.042	−0.075	0.041	0.064

			Group	−8.470	1.188	<0.001	−0.197	0.044	<0.001
RRMS-NON (*n* = 405)	92.03 (11.91)	8.54 (0.42)	Age	−0.080	0.061	0.189	−0.003	0.002	0.094
			Gender	2.933	1.224	0.017	−0.107	0.045	0.019

			Group	−9.951	1.084	<0.001	−0.248	0.039	<0.001
SPMS-NON (*n* = 89)	83.14 (12.07)	8.32 (0.41)	Age	0.139	0.098	0.158	0.003	0.003	0.267
			Gender	−1.807	1.788	0.312	−0.150	0.062	0.015

			Group	−4.253	0.792	<0.001	−0.141	0.027	<0.001
PPMS-NON (*n* = 77)	88.35 (11.31)	8.34 (0.42)	Age	0.037	0.093	0.691	−0.001	0.003	0.654
			Gender	0.802	1.796	0.655	−0.044	0.064	0.492

			Group	N/A	N/A	N/A	N/A	N/A	N/A
HC (*n* = 183)	100.60 (9.41)	8.75 (0.34)	Age	N/A	N/A	N/A	N/A	N/A	N/A
			Gender	N/A	N/A	N/A	N/A	N/A	N/A

**Table 3:** OCT results for the subtypes of MS patients with a history of optic neuritis (ON). Total retinal nerve fiber layer (RNFL) thickness (in *μ*m) and total macular volume (TMV in mm^3^) are given as mean values with standard deviation (SD). ON eyes were compared to ON-non affected eyes of the same MS subtype using generalized estimation equation (GEE) models. GEE models estimate the effect size with standard error (SE) and the respective *P* value.

			GEE models comparing OCT parameters between ON-affected and unaffected eyes of the same subtype						
				RNFL thickness			TMV		
	Total RNFL thickness mean (SD) [*μ*m]	TMV mean (SD) [mm^3^]	Effect	Effect size	SE	*P* value	Effect size	SE	*P* value
			Group	−12.199	1.336	<0.001	−0.237	0.043	<0.001
MS-ON (*n* = 183)	77.88 (14.61)	8.24 (0.45)	Age	−0.147	0.056	0.008	−0.007	0.002	0.001
			Gender	2.989	1.161	0.010	−0.041	0.040	0.299

			Group	−12.859	1.478	<0.001	−0.263	0.048	<0.001
RRMS-ON (*n* = 156)	78.69 (14.91)	8.28 (0.45)	Age	−0.087	0.066	0.185	−0.004	0.002	0.071
			Gender	4.573	1.352	0.001	−0.048	0.046	0.295

			Group	−9.297	2.802	0.001	−0.252	0.100	0.012
SPMS-ON (*n* = 27)	73.19 (11.89)	8.05 (0.41)	Age	0.419	0.216	0.052	0.009	0.008	0.234
			Gender	−7.310	2.591	0.005	−0.250	0.091	0.006

PPMS-ON (*n* = 0)	N/A	N/A	N/A	N/A	N/A	N/A	N/A	N/A	N/A

HC (*n* = 183)	100.60 (9.41)	8.75 (0.34)	N/A	N/A	N/A	N/A	N/A	N/A	N/A

**Table 4:** Differences between MS subtypes without a history of optic neuritis (NON) were analyzed with generalized estimation equations (GEE) models including age, disease duration, and gender as effects.

	GEE models comparing OCT parameters between NON eyes of different MS subtypes						
		RNFL thickness			TMV		
	Effect	Effect size	SE	*P* value	Effect size	SE	*P* value
RRMS-NON versus SPMS-NON	Group	−5.144	1.921	0.007	−0.137	0.066	0.039
	Age	0.062	0.077	0.419	−0.001	0.003	0.775
	Duration	−0.044	0.009	<0.001	−0.001	0.0003	<0.001
	Gender	1.864	1.377	0.176	−0.139	0.051	0.007

RRMS-NON versus PPMS-NON	Group	−1.204	1.022	0.239	−0.104	0.037	0.005
	Age	−0.016	0.073	0.823	−0.002	0.003	0.371
	Duration	−0.034	0.008	<0.001	−0.001	0.0003	<0.001
	Gender	2.785	1.393	0.045	−0.102	0.052	0.051

SPMS-NON versus PPMS-NON	Group	2.634	2.494	0.291	−0.053	0.090	0.553
	Age	0.339	0.175	0.053	0.002	0.006	0.729
	Duration	−0.031	0.011	0.007	−0.001	0.0004	0.224
	Gender	−3.932	2.287	0.086	−0.127	0.086	0.139

**Table 5:** Generalized estimation equation (GEE) models correlating disease duration with RNFL thickness and TMV, respectively. The yearly change based on the effect sizes of the respective GEE model.

	RNFL thickness				TMV			
	Effect size	SE	*P* value	Change per year (*μ*m)	Effect size	SE	*P* value	Change per year (mm³)
All MS-NON	−0.0444	0.0068	<0.001	−**0**.**533**	−0.0012	0.0002	<0.001	−**0**.**014**
RRMS-NON	−0.0413	0.0088	<0.001	−**0**.**495**	−0.0013	0.0003	<0.001	−**0**.**016**
SPMS-NON	−0.0387	0.0174	0.026	−**0**.**464**	−0.0001	0.0006	0.838	−**0**.**002**
PPMS-NON	−0.0088	0.0151	0.562	−**0**.**105**	−0.0009	0.0006	0.131	−**0**.**011**
